# Cold atmospheric plasma for bacterial inactivation in Nile water and wastewater

**DOI:** 10.1038/s41598-026-52839-3

**Published:** 2026-05-20

**Authors:** F. M. El-Hossary, E. A. Noureldein, M. Abo El-Kassem, Aly E. Abo-Amer

**Affiliations:** 1 Physics Department, Faculty of Science, Sohag University, Sohag, 82524 Egypt; 2 Physics department, Faculty of Education and Arts, Sohar University, Sohar 311, Oman; 3 Department of Botany and Microbiology, Faculty of Science, Sohag University, Sohag, 82524 Egypt

**Keywords:** Cold atmospheric plasma (CAP), Corona discharge, Bacterial inactivation, Nile water, Wastewater treatment, Environmental sciences, Microbiology, Water resources

## Abstract

This study aims to evaluate the efficiency of cold atmospheric plasma (CAP) generated using a corona discharge system in inactivating Gram-positive (*Bacillus species*) and Gram-negative (*Escherichia coli*) bacteria in Nile River water and wastewater. CAP was characterized using voltage-current curves and optical emission spectra (OES), while scanning electron microscopy (SEM), growth curve methods, and viable plate counts were used to evaluate antibacterial activity. CAP treatment successfully reduced bacterial counts in both Nile water and wastewater samples, with a reduction of ≥ 6-log in bacterial population after 8 min of treatment in Nile water, and a reduction of ≥ 2.6-log after 6 min of treatment in wastewater samples. Plasma treatment significantly altered bacterial growth kinetics and caused extensive morphological damage. The treated water samples showed a notable change in their physicochemical characteristics, including a moderate rise in electrical conductivity and a reduction in pH. The OES showed strong emission from the second positive system of nitrogen, with minor emission from N₂⁺ and OH radicals, suggesting strong plasma-water interactions. CAP treatment was non-thermal, based on the temperature readings, thus suggesting that the CAP could be used to inactivate bacteria in the Nile River water and wastewater samples in an environmentally friendly way.

## Introduction

All forms of life on Earth are sustained by the provision of water, which is abundant and essential. However, the quality of water is increasingly under threat of microbial contamination, which is a significant global public health problem. Waterborne diseases, including diarrheal disorders, cholera, typhoid fever, hepatitis A, and poliomyelitis, have a strong association with contaminated water sources and poor sanitation facilities^1^. Surface waters, including rivers, are very vulnerable to microbial contamination by the continuous influx of untreated wastewater generated from human settlements, industries, and agriculture. The microbial flora of wastewater is a complex and dynamic ecosystem that includes a variety of pathogenic and non-pathogenic microorganisms. In addition, wastewater is considered a major reservoir for enteric bacteria, including *Salmonella typhimurium* and *Escherichia coli* (*E. coli*)^3–5^. The presence of microorganisms in surface waters significantly increases the risk of disease transmission. Therefore, the disinfection of water is a significant aspect that needs to be addressed. Among the pathogenic microorganisms found in contaminated waters, *E. coli* is commonly used to indicate the presence of fecal contamination in water sources due to their prevalence, persistence, and strong association with the presence of pathogenic microorganisms. In contrast, Gram-positive bacteria, including *Bacillus sp.*, have a high resistance to conventional disinfection methods owing to the formation of endospores and the presence of a thick peptidoglycan cell wall.

Traditional disinfection techniques like ultraviolet light, ozonation, and chlorination have commonly been employed for water disinfection purposes. Nevertheless, these techniques are also accompanied by certain limitations. Chlorination is the most commonly used disinfection method but is known to react with natural organic matter, resulting in the formation of toxic disinfection by-products that are harmful to the environment as well as human health^10^. Furthermore, long-term use of chlorinated disinfectants also promotes the survival of chlorine-resistant bacteria strains^11,12^. Similarly, ozonation is an effective disinfection method but is limited by high operational and maintenance costs, as well as the potential for microbial regrowth in water systems^13^. All these limitations have led to a search for alternative disinfection techniques that are not only effective but also environmentally friendly and chemical-free.

Plasma disinfection is a new disinfection technology that is being explored for application purposes in the environmental as well as biomedical sciences. Initial experiments have shown that bacteria like Gram-positive and Gram-negative bacteria have successfully been inactivated using different types of plasma discharges^14–18^. Cold atmospheric plasma (CAP) has gained substantial interest as a non-thermal method of sterilization. It does not require any harmful chemical reagents and does not involve high temperatures. In addition, using air instead of noble gases makes this method a more convenient and advantageous approach in practice^19^. As air plasma systems eliminate the need for external gas supply and enhance in situ generation of reactive oxygen and nitrogen species (RONS) directly without additives or gas cost, enhancing their applicability for large-scale treatment^51^. However, the effectiveness of liquid-plasma interactions depends critically on the properties of bacteria, including Gram type, strain, and cell density, as well as their physiological states^20^.

A complex of energetic electrons, ions, ultraviolet (UV) photons, electric fields, and RONS in a partially ionized gas is formed in a CAP-treated solution. In this context, reactive species, such as hydrogen peroxide (H₂O₂), superoxide anion (O₂⁻), hydroxyl radical (•OH), and singlet oxygen (¹O₂), are known to play a crucial role in inactivating bacteria by causing oxidative damage to DNA, RNA, and phospholipid membranes in bacteria^22^. Many publications have confirmed the effectiveness of using plasma in removing bacteria in aquatic environments^23–29^, while further studies have investigated the influence of corona discharge plasma on bacterial inactivation^30–33^.

Despite the growing body of knowledge regarding the potential of plasma technology for the inhibition of bacteria, there are knowledge gaps to be addressed. There is a lack of information regarding the influence of plasma technology on the growth dynamics of bacteria, as well as the inactivation mechanism and plasma–water interaction in natural waters. In addition, there is a lack of scientific data regarding the potential use of corona discharge plasma technology for the treatment of Nile water and wastewater.

Previous studies focused mainly on model water or single bacterial strains; research on complex natural matrices like Nile water and raw wastewater is limited^12^. This study examines CAP-induced bacterial inactivation kinetics in those systems by combining microbiological analysis, plasma diagnostics, and physicochemical characterization to clarify the underlying mechanisms.

This research aims to bridge the knowledge gap regarding the application of corona discharge cold atmospheric plasma (CAP) for bacterial inactivation in Nile water and wastewater. Specifically, it evaluates the effectiveness of CAP against both Gram-positive and Gram-negative bacteria in these complex water matrices. It also investigates plasma–water interactions, bacterial growth dynamics following plasma treatment, and plasma-induced morphological changes in bacterial cells.

## Methods

### Experimental corona plasma system

**Fig. 1:**
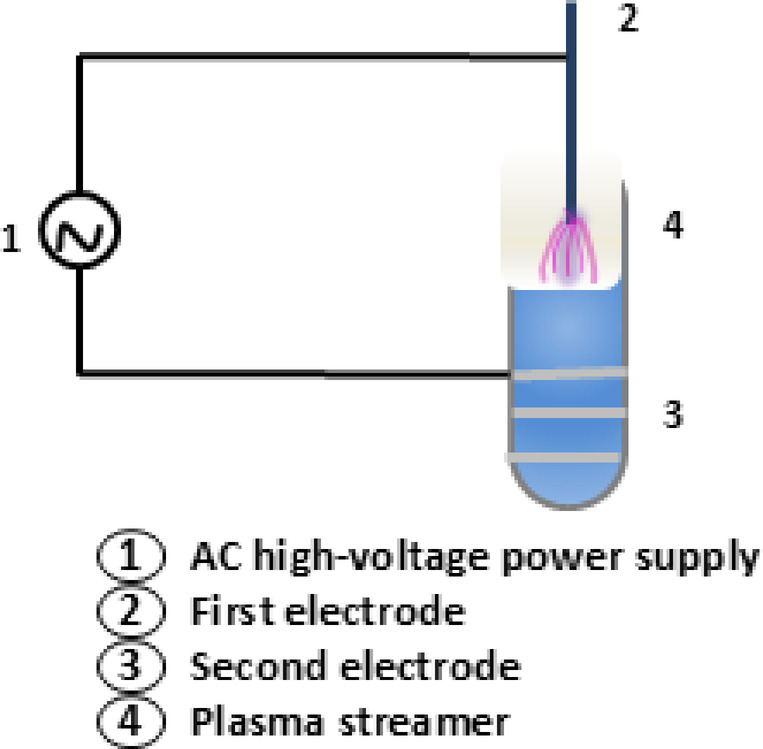
Experimental CAP set-up.

The experimental setup for the corona discharge device is depicted in Fig. 1. The corona discharge apparatus was assembled from a coaxial electrode assembly connected to a high-voltage alternating current power supply (NeonPro NP-7500-30, 7.5 kV, 30 mA). The silver tape anode was wrapped around the outside of the glass tube, while the 0.3 mm stainless-steel wire cathode was situated in the middle of the tube (15 mm inner diameter). The distance between the cathode tip and the water surface was fixed at 5 mm. Plasma treatment was performed at 2.5 kV to ensure stable non-thermal corona discharge and avoid transition to arc discharge at higher voltages, thereby maintaining the desired non-equilibrium plasma conditions^16^.

Under these conditions, plasma appeared as filamentary streamers above the water surface, with charged species drifting toward the anode^34^. Different plasma exposure times were selected according to the experimental objectives. Exposure durations ranging from 2 to 8 min were applied to determine the optimal time required for complete bacterial inactivation in 10 mL water samples. For optical density experiments, a 24-minute exposure was used with 50 mL samples, based on preliminary optimization tests. Regarding agitation, the experiments were conducted under controlled conditions without external stirring to isolate the effect of corona discharge plasma on bacterial inactivation.

### Atmospheric plasma diagnostic

To characterize the plasma discharge, waveforms of voltage and current were recorded. The experimental setup employed in this study was adapted from the design previously reported by Abd El-Reda et al.^35^. The high voltage and current waveforms of the corona plasma were examined using high-voltage probes and current sensors built into the electrical circuit of the plasma system by a two-channel digital oscilloscope (XDS4504). The discharge current was measured using a CP-07 + current probe operated in the 400 mA range, providing a sensitivity of 1 mA/mV. Consequently, the recorded signal from channel 1 was directly converted to discharge current without additional scaling. The applied high voltage was simultaneously measured using a high-voltage probe (1000:1) connected in parallel to a plasma electrode and channel 2. Both voltage and current waveforms were recorded with a temporal resolution of 0.02 µs.

A spectrometer that was attached to an optical cable and placed one centimeter away from the plasma was used to collect optical emission data. Species identification and spectral feature recording in the 300–900 nm region was made possible by this configuration.

### Collection of water samples

Two different natural water sources were investigated; the water samples were gathered in clean, autoclaved bottles. The first sample source was Nile water, collected from the Nile River in Sohag Governorate, Egypt. The second sample source consisted of raw sewage water collected from the Sohag Governorate sewage treatment plant.

### Pour plate methodology

For both water samples, serial dilutions were prepared to determine the optimal dilution factor by the pour plate method. All samples were prepared under strictly controlled and standardized conditions. Specifically, each experiment was conducted using the same volume and concentration of 0.9% saline solution (9 mL), followed by the addition of 1 mL from the same raw water sample to ensure consistency across all tests and minimize variability between experiments.

Five test tubes, each with 10 mL of diluted water from a specific source, were exposed to corona discharge plasma at room temperature for 2, 4, 6, and 8 min, plus an untreated control (0 min). For bacterial enumeration, 1 mL of water was aseptically transferred, before and after plasma treatment, into sterile Petri dishes using the pour plate technique. Nutrient agar and MacConkey agar media were used for Nile water and wastewater samples, respectively. After solidification, the plates were incubated for 24 h at 37 °C. Using the conventional plate count method, the colony-forming units per milliliter (CFU/mL) were used to calculate the number of surviving bacteria. The treated volume was monitored before and after plasma exposure, and no significant volume loss was observed during the treatment time.

### Identification of Gram-positive and Gram-negative bacteria

Gram staining was performed to differentiate bacterial species. *Bacillus* sp. (Gram-positive) appeared as purple rod-shaped bacilli, while *E. coli* (Gram-negative) appeared as pink rod-shaped cells under oil immersion microscopy (100×).

### Bacterial growth curve analysis

In this experiment, plasma was applied to bacterial cells at two distinct growth stages: the early exponential phase and the mid-exponential phase. The aim here is to investigate the impact of plasma treatment on bacterial growth curves and to determine whether plasma exposure leads to complete bacterial lysis, thereby preventing regrowth following incubation. The study also examined whether bacteria in the mid-exponential phase are more or less susceptible to plasma-induced cell lysis compared to bacteria in the early exponential phase. Using a spectrophotometer, turbidity was measured as optical density at 600 nm (OD₆₀₀) to track bacterial growth. Initially, a single *Escherichia coli* colony was inoculated into 5 mL of nutrient broth (NB) medium (3 g yeast extract, 5 g sodium chloride, and 5 g peptone per liter of demineralized water). The colony was then incubated for the entire night to reach the stationary phase. Subsequently, 1 mL aliquots of the overnight culture were transferred into three separate conical flasks, each containing 50 mL of sterile broth medium, as described below:

- Control flask: Cultures were incubated in a shaking incubator without plasma treatment, and optical density (OD) was measured at hourly intervals.
- Early exponential phase treatment: At 0 h, cells were exposed to plasma for 24 min, corresponding to the previously optimized exposure time required for complete inactivation in 50 mL suspensions. Optical density was measured immediately after treatment and subsequently monitored hourly during incubation.
- Mid-exponential phase treatment: Cultures were first incubated for 4 h to reach the mid-exponential phase and then exposed to plasma for 24 min. Optical density was recorded immediately after treatment and at hourly intervals during subsequent incubation.

### Physicochemical characterization of treated water

A mercury thermometer (Testo-T1) was used to monitor the temperatures of the plasma-treated water at various points in time. The thermometer is submerged in the water tube, and the temperature is recorded at three separate points within the tube: the center of the water, 1 cm below the surface, and on the surface of the water exposed to plasma. The same technique is used to test the temperature of the untreated control sample. To examine and track the change, the recorded temperature readings were tabulated.

To look into the impact of atmospheric corona discharge treatment on pH value of the treated water, a calibrated pH meter (model: Adwa AD1030, Romania) with a glass electrode to detect acidity or alkalinity was used to test the pH values of the untreated and treated each water type, Nile water and wastewater, at intervals of 2, 4, 6, and 8 min. Before measurement, the pH meter is calibrated using standard buffer solutions for accuracy purposes. The samples are mixed by gentle stirring to ensure homogeneity. The pH probe is inserted into the water, and a measurement is taken after the pH meter stabilizes. An untreated water control sample is also taken for measurement. The pH probe is cleaned with distilled water before measurement to avoid cross-contamination. The effect of plasma therapy on the chemical properties of the water is evaluated by comparing the pH readings.

A conductivity/TDS meter for measurement is a tabletop conductivity/TDS meter (Model: Adwa AD3000, Romania). Electrical conductivity (EC) is measured for samples of Nile water and effluent before and after treatment using this meter. The samples are not immediately measured after plasma treatment, as this effect might alter conductivity readings. All samples are left to cool to room temperature before measurement, as a precautionary measure against potential effects of plasma-induced heating on conductivity readings. The temperature of each sample is recorded before measurement, and it is noticed that all samples reach approximately the same temperature. The probe is cleaned with distilled water before measurement to avoid cross-contamination. The EC readings are recorded after stable readings are obtained.

### Morphological analysis

Treated and untreated bacterial suspensions were centrifuged using a centrifuge operating at 4,000 rpm for 15 min and then washed with a 0.9% saline solution. The collected cells, also known as pellets, were chemically fixed using a 90% ethanol solution, dried with air, and then coated with a thin gold film before examination. Scanning Electron Microscopy analysis was carried out using a JSM-5400 LV microscope for *Bacillus* sp. (15 kV accelerating voltage, 15,000–20,000x magnification) and a Sigma 500 VP microscope for *Escherichia coli* (5 kV accelerating voltage, 7,500x magnification). Multiple regions from each sample were examined to ensure representative morphological observations.

### Statistical analysis

The statistical analysis was carried out using IBM SPSS Statistics program (version 24). All experiments were conducted using independent measurements. Data is expressed as mean ± standard error (SE). The standard deviation (SD) was calculated, and the SE was determined as SD/√n, where n represents the number of independent measurements. Error bars in all graphical representations indicate SE. Prior to analysis, data were examined for consistency and suitability for parametric testing. Statistical comparisons were carried out using one way analysis of variance (ANOVA) and paired-samples t-test, as appropriate to the experimental design. At *p* < 0.05, differences were deemed statistically significant, and at *p* < 0.01 or *p* < 0.001, they were deemed extremely significant.

## Results

### Voltage/current waveforms and OES analysis

**Fig. 2:**
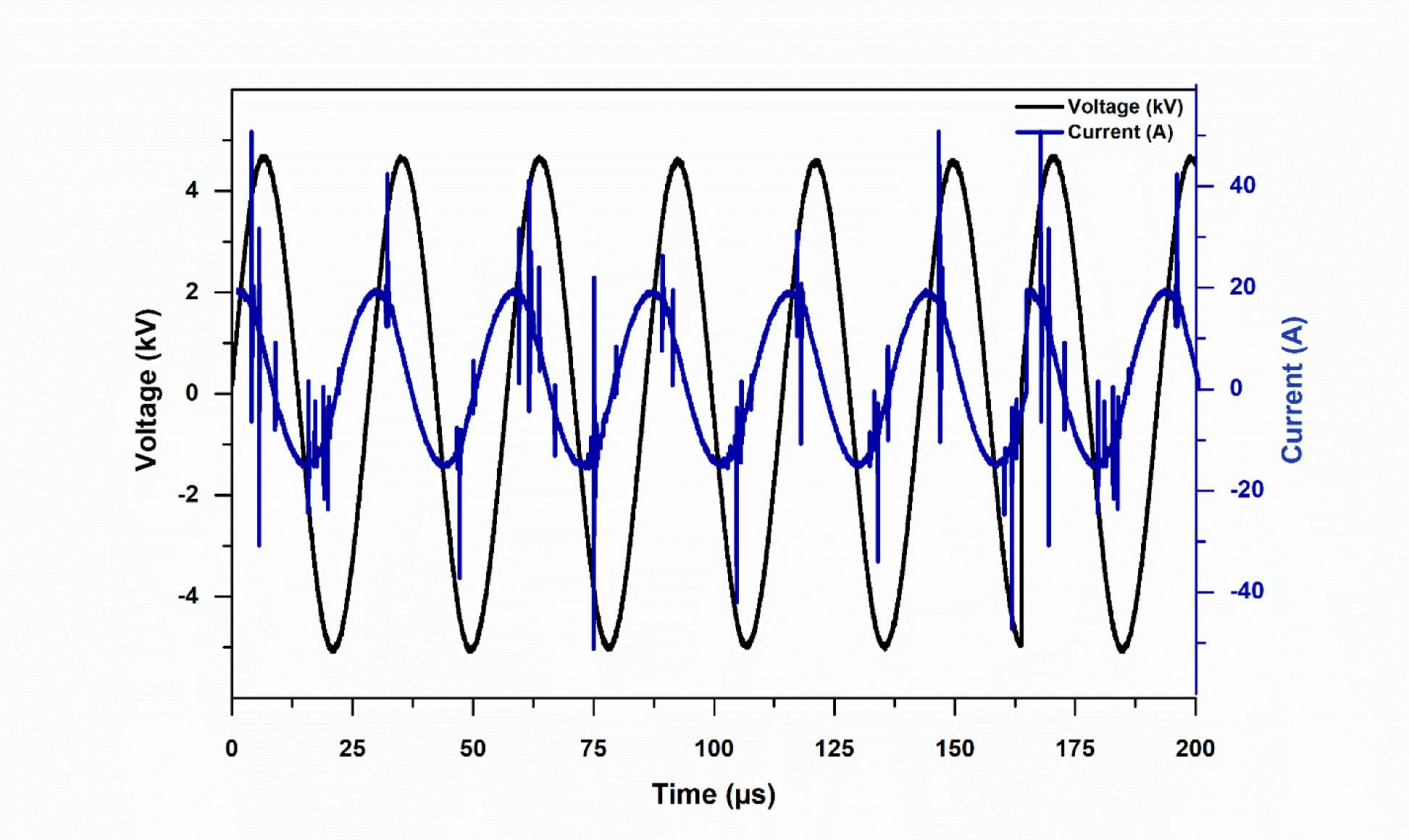
High voltage and current waveforms of the CAP system at 2.5 kV applied voltage.

Time-resolved voltage and current waveforms of the corona discharge generated above the water’s surface at an applied voltage of 2.5 kV are shown in Fig. 2. The discharge current (blue curve) is marked by impulsive current spikes connected to brief streamer events that happen close to the voltage peaks, whilst the applied voltage (black curve) shows stable sinusoidal behavior. These characteristics can be linked to variations in electron and ion transport pathways.

Under plasma exposure, the water surface serves as a conductive, polarizable interface capable of sustaining significant interfacial charge accumulation. The origin, propagation, and quenching of successive streamer micro-discharges are known to be affected by this surface charge’s distortion of the local electric field near the interface^36^. Reactive oxygen and nitrogen species (RONS) are encouraged to form because the discharge, being a non-thermal (non-equilibrium) plasma, usually keeps the gas temperature low while maintaining energetic electrons^37^. Plasma-based water treatment can be supported by repeating streamer activity above the water’s surface, which can improve plasma–liquid interaction and make it easier for reactive species to enter the liquid phase^38^.

**Fig. 3:**
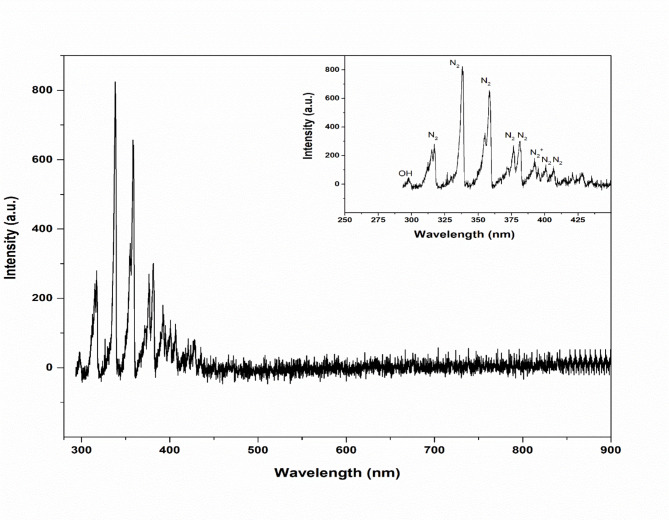
Optical emission spectrum of CAP generated in the tube at 2.5 kV. The main figure shows the emission spectrum in the wavelength range of 200–900 nm. The inset presents a magnified view of the UV region (250–450 nm).

The optical emission spectrum (OES) of the air plasma produced inside the plasma reactor, obtained throughout the wavelength range of 200–900 nm, is displayed in Fig. 3. One well-known characteristic of air plasma discharges is that strong bands in the 300–400 nm range dominate the OES^39,40^. The second positive system (SPS) of molecular nitrogen N_2_ is primarily responsible for these bands^41,42^. At approximately 317, 337.8, 358.3, 376.8, and 381.5 nm, distinctive N₂ emission bands are seen. Molecular nitrogen excitation is the predominant radiative mechanism in plasma, as seen by the maximum intensity band at 337.8 nm. The first negative system (FNS) of ionized nitrogen N_2_^+^ is shown by a clear emission line at 392.4 nm, which indicates the presence of high-energy electrons and effective ionization processes within the discharge^39,40^. The OH radical, which is frequently linked to water vapor or ambient humidity in air plasmas, is responsible for the feeble emission observed close to 296.7 nm^40,43^. The emission intensity significantly drops at wavelengths greater than 400 nm, which is in line with the restricted number of permitted electronic transitions of air plasma species in the visible range^39,41^.

### Effect of CAP on viable bacterial count

**Fig. 4:**
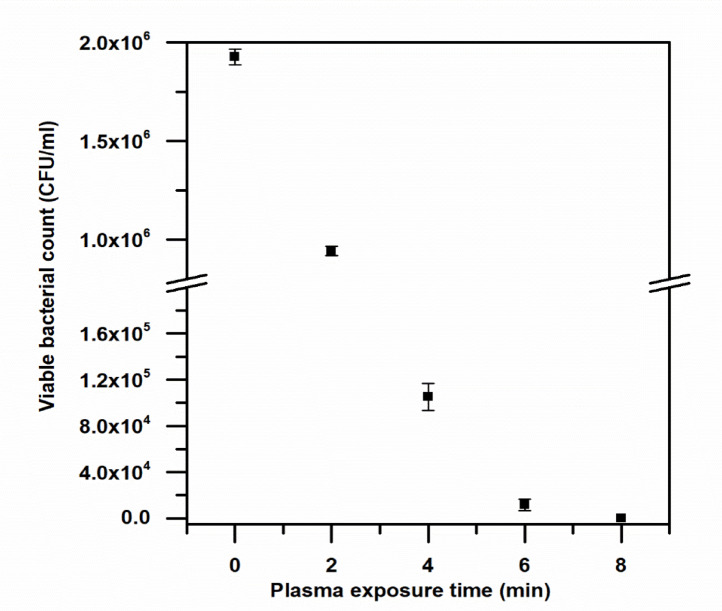
Bacterial colonies number in Nile water with time of CAP treatment (0–8 min).

**Fig. 5:**
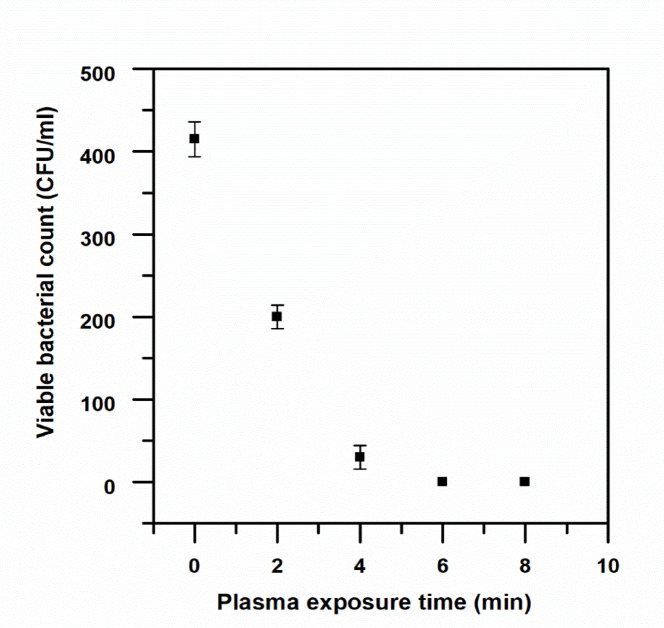
Bacterial colonies number in wastewater with time of CAP treatment (0–8 min).

The plate count method was employed to quantify the number of surviving colony-forming units per milliliter (CFU/mL) following plasma treatment. Figure 4 illustrates a clear decrease in the number of viable bacteria in Nile water with an increase in the time of plasma treatment, with no colonies being observed after 8 min of treatment. Similarly, Fig. 5 indicates a clear decrease in the number of viable bacteria in wastewater samples with an increase in the time of treatment, with complete inactivation being observed after 6 min of treatment. Gram-negative bacteria were more susceptible to treatment with plasma, which could be attributed to the susceptibility of the outer membrane of Gram-negative bacteria to damage by reactive species, thereby resulting in rapid disruption of their envelopes. Gram-positive bacteria were more resistant to treatment with plasma, which could be attributed to the thicker peptidoglycan layer in Gram-positive bacteria, although complete structural disruption was observed with prolonged treatment.

### Scanning electron microscopy analysis

**Fig. 6:**
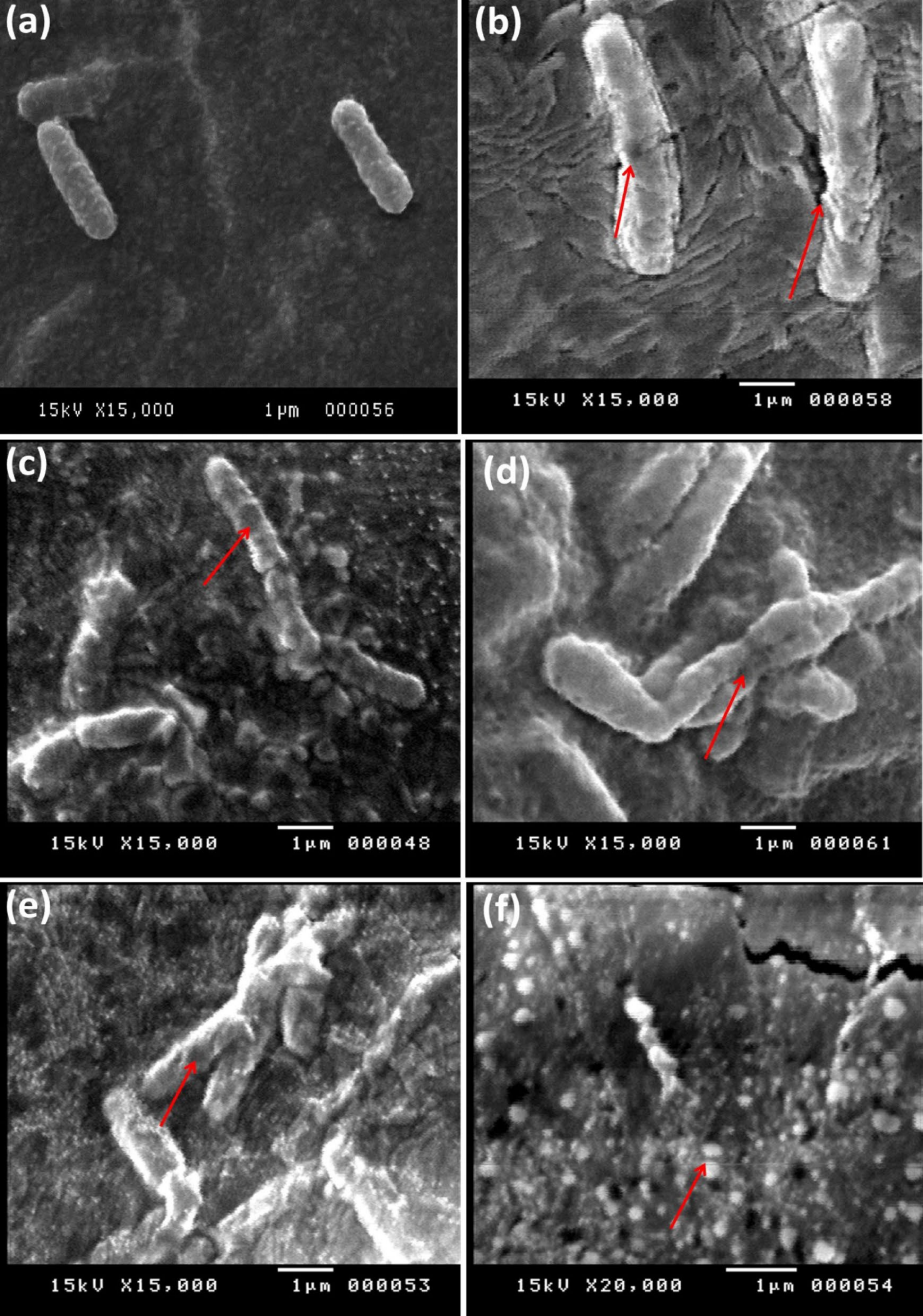
Bacillus sp. cell morphology: **a** untreated control; **b** 2 min; **c** 4 min; **d** 6 min; **e** 8 min; **f** higher magnification of 8 min. Red arrows indicate deformed or damaged cells.

The shape changes are visible under SEM for *Bacillus* cells that are exposed to CAP. These changes are quite distinct from those of untreated controls. While the untreated cells retain their normal rod-like shape and smooth surface integuments (Fig. 6a). In contrast, CAP-treated cells (Fig. 6b–e) exhibited pronounced structural damage. After 2 min of treatment (Fig. 6b), cells appeared visibly deformed, with irregular shapes and roughened surfaces, indicating membrane damage.

As the exposure time increases (see Fig. 6c, d, and e), however, the cells lose their uniformity in size and shape, manifesting surface dimples and small pores as the cell envelopes start to break down gradually. A closer view of the cells exposed to the radiation for 8 min (see Fig. 6f), which is of high magnification, shows that very few badly shrunk and damaged cells are left, which indicates high levels of inactivation.

**Fig. 7:**
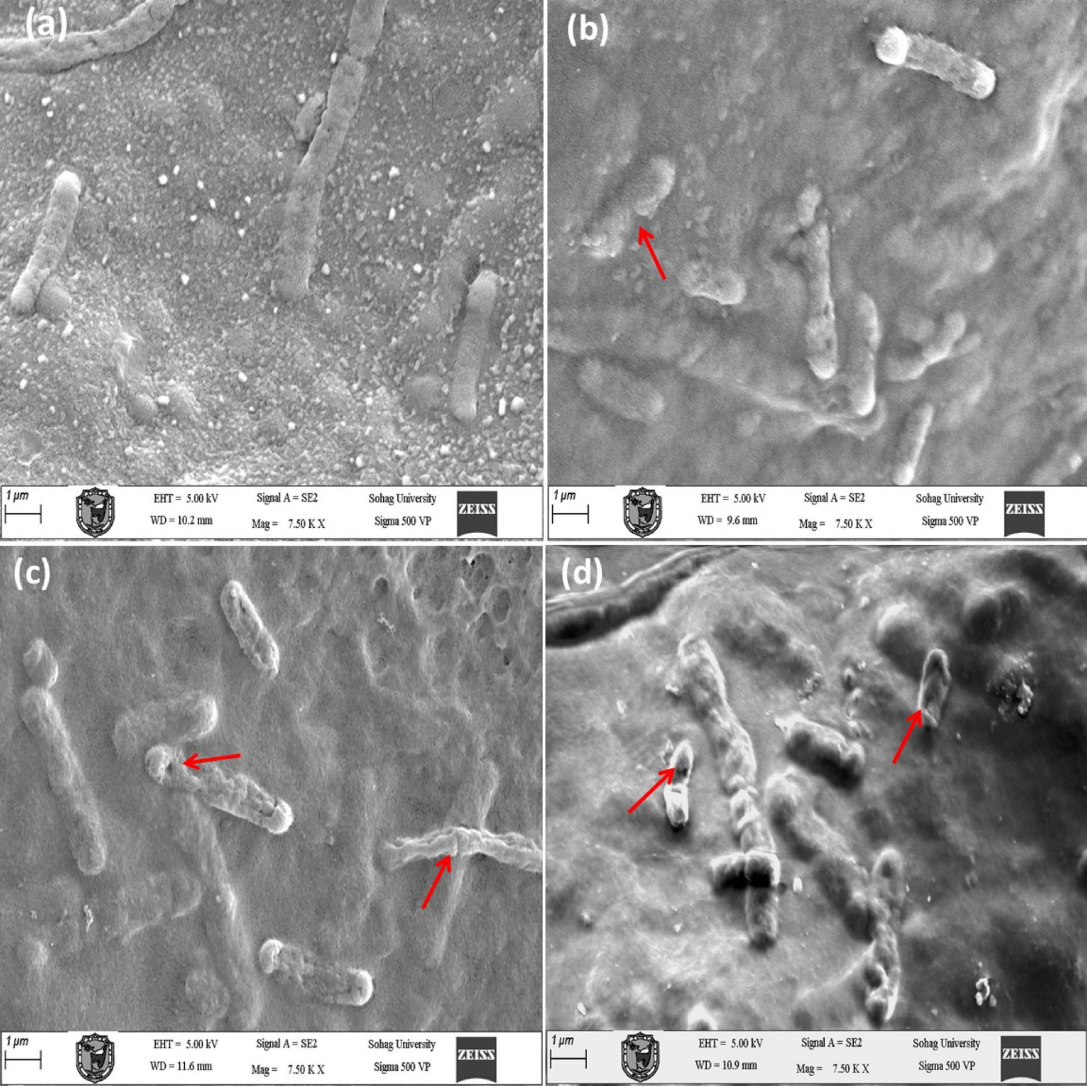
SEM images of E. coli: **a** before plasma; **b** after 2 min; **c** after 4 min; **d** after 6 min. Red arrows indicate deformed or damaged cells.

E. coli cells treated with CAP have a similar effect on the cells, showing significant damage with the SEM images (Fig. 7). The walls of the cells are damaged, with the surface becoming rough and having an irregular shape. When the treatment time increases to 4 and 6 min (Fig. 7b–d), the cells take an irregular shape with the formation of dimples and the appearance of pores, indicating significant damage to the cells due to the plasma action.

### Effect of plasma treatment on bacterial growth curve

The bacterial growth curve typically has four phases: lag, exponential, stationary, and death phases, which represent the dynamic balance between anabolic and catabolic processes in the bacterial culture^44^. During the lag phase, the bacterial culture adjusts to the new environment and accumulates the necessary enzymes for the growth process to continue. When the bacterial culture is transferred from one medium to another identical medium, the lag phase can be eliminated, and the exponential phase can be reached immediately.

In the exponential phase, the bacterial culture divides rapidly and exponentially with a constant generation time. During this phase, the logarithm of the bacterial number is directly proportional to the time, i.e., the logarithm of the bacterial number is linearly related to the time^45^. After the exponential phase, the stationary phase is reached, where the nutrients in the medium become depleted, and the metabolic wastes accumulate, leading to a balance between the death and growth rates in the bacterial culture. Finally, in the death phase, the nutrient depletion in the medium causes the death rate to exceed the growth rate, leading to a decline in the bacterial population^46^.

Optical density at 600 nm (OD600) is an indirect method of determining the concentration of bacteria in a liquid culture by measuring the amount of light scattered by the suspended bacteria, as opposed to the amount of light absorbed by the bacteria. An increase in OD600 indicates an increase in the concentration of suspended bacteria, while a corresponding decrease in OD600 following the application of the plasma treatment indicates a corresponding decrease in suspended bacterial matter.

**Fig. 8:**
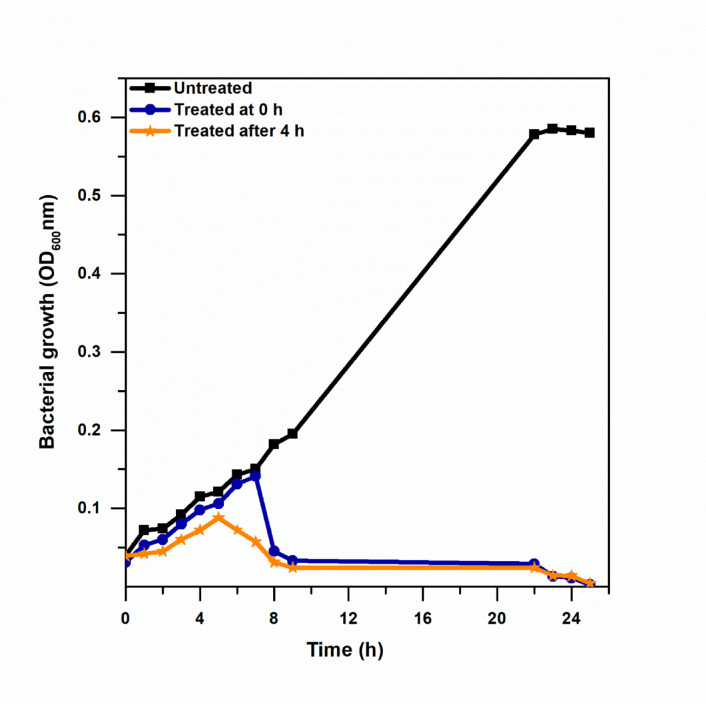
Bacterial growth curve (OD_600_) with distinct phases over 24 h.

As shown in Fig. 8, the untreated control sample exhibited the typical growth curve of a bacterial culture, with the OD600 increasing as the culture entered the exponential phase of growth and levelling off as the culture entered the stationary phase of growth. As shown in the second sample, the application of plasma treatment at the beginning of the exponential phase of growth resulted in an immediate decrease in OD600, indicating the lysis and subsequent inactivation of a portion of the bacterial culture. As the culture grew, the surviving bacteria began to divide, causing a corresponding increase in OD600. As the effects of the plasma treatment accumulated, the lysis of the bacterial culture dominated the culture, causing the OD600 to subsequently decrease.

In the third sample, initial incubation without plasma exposure allowed normal exponential growth. When plasma treatment was applied during the mid-exponential phase, partial lysis and cellular damage were observed, as reflected by a decrease in OD. After approximately one hour, limited regrowth of surviving cells produced a brief OD increase, followed by a progressive decline as damaged and lysed cells became dominant.

### Temperature, pH, and EC of treated water

**Fig. 9:**
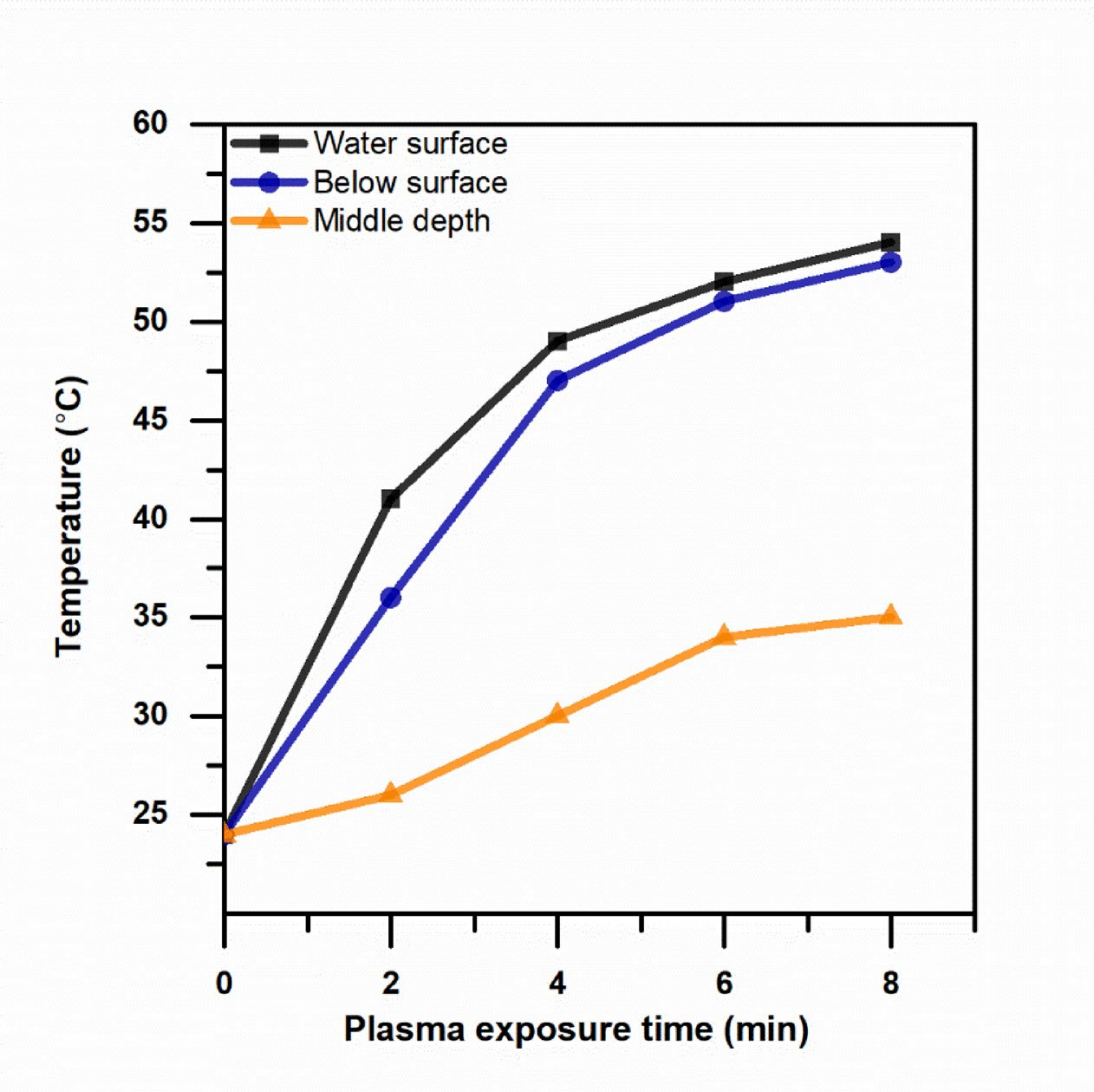
The relationship between the water temperature at various places and the plasma exposure duration.

Figure 9 demonstrates that the water temperature increased with plasma treatment time, reaching a maximum of approximately 54 °C at the plasma-exposed surface and gradually decreasing with increasing water depth. Since bacterial inactivation typically requires temperatures exceeding 65 °C, and many pathogenic microorganisms require temperatures above 74 °C^49^, the recorded temperature values indicate that the applied CAP treatment operated under non-thermal conditions. Therefore, thermal effects were unlikely to be the primary mechanism responsible for bacterial inactivation observed in this study.

**Fig. 10:**
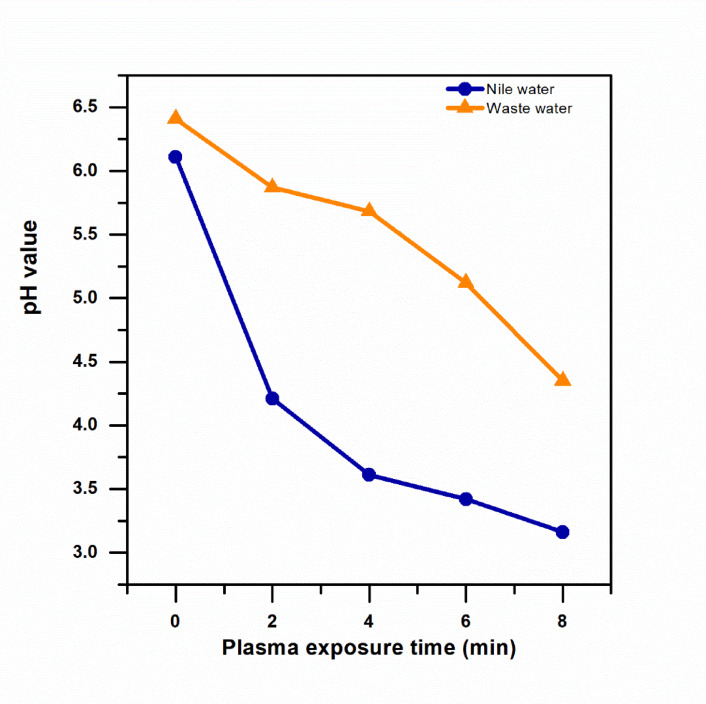
Variation of pH of Nile water and wastewater samples as a function of plasma treatment time.

The initial pH of Nile water and wastewater samples was 6.44 and 6.21, respectively, as measured using a calibrated pH meter before plasma treatment. The pH gradually decreased with increasing plasma treatment time, reaching a value of 3.11 after 8 min of treatment, as shown in Fig. 10. This data agrees with the reported by^35^ which employed the same method. The low pH level of the water has been attributed to the effect of the plasma treatment process, which promotes the formation of compounds that enhance the solution’s acidity levels. These include nitric acid, which results from the interaction of nitrate radicals with hydrogen produced by the dissociation of water due to the effect of the plasma treatment process. Solutions with low pH are effective in controlling the activity of disease-causing organisms^50^. However, the rate of pH reduction was higher in Nile water than in wastewater after the same time of exposure to the plasma treatment process.

The higher rate of pH reduction in Nile water can be attributed to the lower buffering capacity of the water. The effect of the plasma treatment process was found to produce acidic compounds such as nitric and nitrous acids in the water due to the interaction of the water with the atmospheric air during the treatment process^51,52^. On the other hand, the wastewater contains higher concentrations of bicarbonates, organic matter, and ammonia that counteract the effect of the formation of the acidic compounds by the plasma treatment process^53^.

**Fig. 11:**
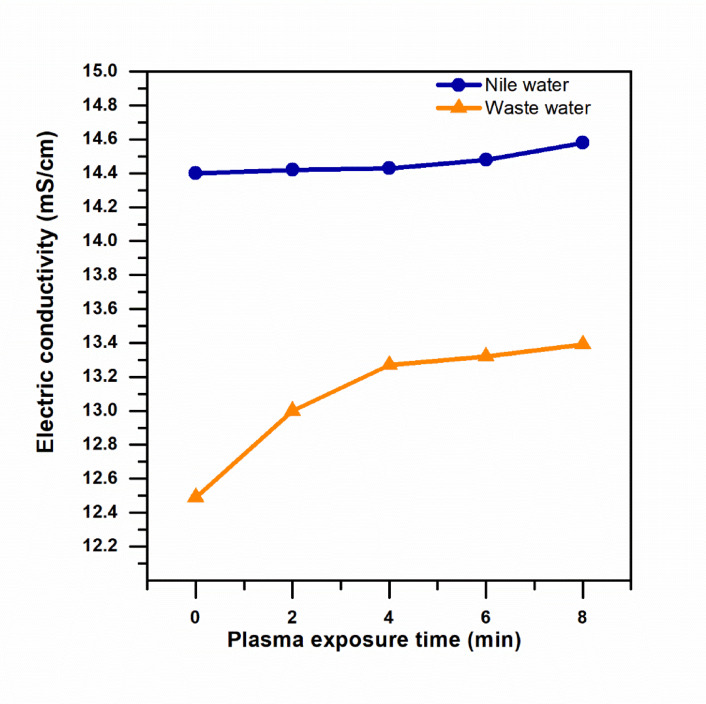
EC variation in Nile water and wastewater samples as a function of plasma treatment duration.

Figure 11 shows that the electrical conductivity of Nile water and wastewater increases due to the effect of the plasma treatment process with the longer period of exposure to the treatment process. However, the rate of increase of the electrical conductivity of the wastewater was higher than that of the Nile water due to the higher concentration of organic matter and ions in the wastewater. The effect of the plasma treatment process promotes the formation of various ions such as NO₃⁻, NO₂⁻, H⁺, and H₂O₂ that significantly enhance the electrical conductivity of the water.

### Statistical evaluation

As shown in Tables 1 and 2, a statistically significant negative correlation is seen for the duration of plasma treatment and bacterial colony counts. The one-way analysis of variance for this experiment showed that *p* = 0.0001 (*p* < 0.001), indicating a highly significant effect for plasma treatment on bacterial colony counts for both water samples. The paired samples t-test showed that there is a statistically significant reduction in bacterial colony counts for all durations of plasma treatment when compared to the untreated control group (*p* < 0.05).

**Table 1:** Statistical analysis of Nile water samples using one-way ANOVA and paired-samples t-test.

Source of variation	Sum of squares	df	F-value	ρ (*p*-value)	Significance
Between Groups (Time)	4.76 × 10^12^	3	1378.15	0.0001	Significant
Within Groups (Error)	4.61 × 10^9^	4	—	—	—
Total	4.77 × 10^12^	7	—	—	—

Time (min)	Mean CFU/ml (± SD)	Comparison vs. 0 min	t-value	ρ (p-value)	Significance
0	1.93 × 10⁶ ± 5.66 × 10⁴	—	—	—	Control
2	9.43 × 10⁵ ± 3.30 × 10⁴	0 vs. 2 min	15.526	0.041	Significant
4	1.05 × 10⁵ ± 1.65 × 10⁴	0 vs. 4 min	64.294	0.010	Significant
6	1.17 × 10⁴ ± 7.07 × 10³	0 vs. 6 min	54.714	0.012	Significant
8	0 ± 0	0 vs. 8 min	48.167	0.013	Significant

**Table 2:** One-way ANOVA and paired-samples t-test in a statistical study of wastewater samples.

Source of variation	Sum of squares	df	F-value	ρ (*p*-value)	Significance
Between Groups (Time)	218,237	3	342.33	0.0001	Significant
Within Groups (Error)	850	4	—	—	—
Total	219,087	7	—	—	—

Time (min)	Mean CFU/ml (± SD)	Comparison vs. 0 min	t-value	ρ (p-value)	Significance
0	415 ± 21.2	—	—	—	Control
2	200 ± 14.14	0 vs. 2 min	43	0.015	Significant
4	30 ± 14.14	0 vs. 4 min	15.41	0.041	Significant
6	0 ± 0	0 vs. 8 min	27.66	0.023	Significant

## Discussion

The results suggest that CAP, which was generated using a corona discharge, is very effective in reducing the population of both Gram-positive bacteria, *Bacillus sp*., and Gram-negative bacteria, *E. coli*, in Nile River water and wastewater. The significant reduction in the number of colony-forming units per milliliter (CFU/mL) confirms that exposure to non-thermal plasma plays an essential role in bacterial inactivation. The antibacterial effects can be attributed to the interaction of the CAP with water, which leads to the formation of reactive oxygen and nitrogen species (RONS). Although the interaction of the CAP with water and the formation of reactive species have been observed during the degradation of organic compounds using a corona discharge and DBD plasma, such as atrazine degradation in water, the study by Papalexopoulou et al. showed the importance of the role of plasma-induced chemistry in liquid phase treatment processes^54^, our study expands these ideas to include biologically complex systems, where bacterial inactivation depends on bacterial growth, bacterial responses, and structural damage, thereby emphasizing the complexity of bacterial control using CAP. Moreover, the results of electrical characterization and optical emission spectroscopy (OES) analysis also support the generation of reactive species such as hydroxyl radicals (•OH), nitrite (NO₂⁻), nitrate (NO₃⁻), and hydrogen peroxide (H₂O₂), which are known to cause oxidative stress and damage to the essential components of the bacterial cell, such as DNA, proteins, and lipid membranes^55,56^. The dominance of nitrogen-containing species in the plasma emission spectra is consistent with the results of earlier studies on the performance of APS systems, in which nitrogen-containing species play a role in the generation of long-lived reactive species in water^46^.

The results also show that Gram-negative bacteria such as *E. coli* are more susceptible to CAP treatment than Gram-positive bacteria such as *Bacillus sp.* This difference in susceptibility can be attributed to the differences in the composition of the bacterial envelope. Gram-positive bacteria have a thick peptidoglycan layer in their envelope, which provides mechanical stiffness and some degree of protection to the bacteria. Gram-negative bacteria, on the other hand, have a thin peptidoglycan layer and an outer lipid layer in their envelope, which can be subject to lipid peroxidation and oxidative damage^47^.

The difference in inactivation efficiency, ≥ 6-log reduction in Nile water and ≥ 2.6-log reduction in wastewater, can be attributed to matrix effects, including higher organic load and buffering capacity that reduce reactive species activity^26,52^, as well as the use of selective MacConkey agar, which may underestimate the initial bacterial load compared to the general nutrient agar used for Nile water.

The results of the scanning electron microscopy (SEM) analysis provide direct evidence of the progression of the morphological damage to the bacterial cells, as shown by the roughening of the surface, depressions, perforations, and collapse of the cell wall. These effects are characteristic of the oxidative degradation of the bacterial envelope. These results are consistent with earlier findings suggesting that reactive oxygen species are capable of oxidizing the side chain groups of amino acids, thereby causing the denaturation of proteins^57^.

The timing of the application of the CAP treatment with respect to the phase of the bacterial culture growth cycle was also seen to influence the efficiency of the treatment. More efficient inactivation was observed with the mid-exponential phase of the culture cycle as opposed to the early exponential phase. Cells are more susceptible to oxidative stress and electrophysical damage during the mid-exponential phase of the culture cycle as a result of increased metabolic activity, as well as the suppression of the activation of the stress response system^46^. The transient increase observed in the OD600 after the CAP treatment indicates the partial survival of the treated culture, as well as the ability of the damaged cells to regenerate to a certain extent. However, the subsequent decline of the OD600 indicates irreversible damage to the bacterial culture.

Significant physicochemical changes were observed to the treated water as a result of the application of the CAP treatment. A significant reduction of the pH of the treated water to a value of 3.1 was observed, as well as an increase in the electrical conductivity of the treated water. Previous studies have shown that plasma-treated water retains its antimicrobial activity even after pH neutralization, confirming that reactive species, rather than acidity alone, play the dominant role in bacterial inactivation^59^.

These results are consistent with the production of PAW with high levels of ROS. The greater extent of the reduction of the pH of the treated Nile water with respect to the wastewater is a result of the relatively lower buffering capacity of the former. These physicochemical effects are significant as they contribute to the inactivation of the bacterial culture. Importantly, the results of the measurements of the temperature of the treated culture confirmed that the inactivation of the culture was not the result of the heat effects of the CAP treatment.

The findings obtained in this research correlate with existing studies on plasma-mediated microbial inactivation in aqueous environments. In these environments, the bactericidal effect of plasmas is explained by the action of reactive species, UV radiation, and temporary electric fields. This research extends existing knowledge by investigating bacterial growth patterns under plasma treatment and determining that bacteria are more susceptible to this treatment during the mid-exponential phase of growth. The application of cold atmospheric plasma (CAP) to Nile River water and wastewater samples fills a significant gap in existing knowledge. Only a limited number of studies have examined CAP application to aqueous environments with complicated chemical compositions. The findings obtained in this research support CAP as a promising and eco-friendly substitute for traditional disinfection methods such as chlorination and ozonization. Unlike traditional disinfectants used for water treatment, CAP operates at normal pressure and near room temperature. It does not produce harmful disinfection by products. CAP’s effectiveness against resistant bacterial species such as *Bacillus sp.* indicates its potential for future application in sustainable water and wastewater treatment systems. This is especially true for regions that face persistent challenges with microbial contaminants.

## Conclusion

The purpose of this study is to assess the application of cold atmospheric plasma (CAP) created by corona discharge for bacterial inactivation in Nile River water and wastewater. The electrophysical characteristics and optical emission spectroscopy (OES) indicate that there are significant interactions between plasma and water. This allows for the generation of reactive oxygen and nitrogen species. This study indicates that CAP application causes a significant reduction in bacterial populations. The study found that Gram-negative bacteria are more susceptible to CAP applications compared to Gram-positive bacteria. This effect is due to differences in membrane structure. The growth curve indicates that bacterial lysis plays a major role in CAP application. This effect is pronounced when CAP application occurs at the exponential phase. The scanning electron microscopy (SEM) images indicate that CAP applications cause significant morphological changes. The evaluation of physicochemical parameters further confirmed the non-thermal nature of the applied CAP, as evidenced by moderate temperature increases accompanied by significant reductions in pH and increases in electrical conductivity, reflecting plasma-induced chemical modifications of the treated water. Overall, the findings highlight corona discharge CAP as a promising, environmentally friendly, and effective strategy for mitigating microbial contamination in natural water bodies and wastewater treatment applications.

## Data Availability

The data supporting the findings of this study are available from the corresponding author upon reasonable request.
